# Harnessing Transient Expression Systems with Plant Viral Vectors for the Production of Biopharmaceuticals in *Nicotiana benthamiana*

**DOI:** 10.3390/ijms26125510

**Published:** 2025-06-09

**Authors:** Sayed Abdul Akher, Kevin Yueju Wang, Kylie Hall, Oluwaseyi Setonji Hunpatin, Muhammad Shan, Zenglin Zhang, Yongfeng Guo

**Affiliations:** 1 Tobacco Research Institute, Chinese Academy of Agricultural Sciences, Qingdao 266101, China; 2 Graduate School of Chinese Academy of Agricultural Sciences, Beijing 100081, China; 3 Division of Math and Natural Sciences, University of Pikeville, Pikeville, KY 41051, USA

**Keywords:** plant molecular farming, transient expression, *N. benthamiana*, recombinant proteins, biopharmaceuticals, controlled environment agriculture

## Abstract

Plant Molecular Farming (PMF) capitalizes on the unique properties of plants as bioreactors to efficiently produce valuable proteins, pharmaceuticals, and enzymes. This review emphasizes the critical role of transient expression systems, particularly in *Nicotiana benthamiana*, due to its susceptibility to various pathogens. Viral vector-based transient expression has proven essential during health emergencies like COVID-19, enabling rapid recombinant protein production. The review also evaluates different transient expression platforms and highlights their applications in biopharmaceutical production, education, synthetic biology, and gene editing. Advances in viral vector modification, hydroponics, and Controlled Environment Agriculture (CEA) are presented as transformative innovations enhancing scalability and regulatory compliance. Furthermore, glycoengineering advancements broaden the range of producible biopharmaceuticals, improving global medication access. By exploring these advancements, this review underscores the vast potential of transient expression systems to meet dynamic scientific and market demands, positioning PMF as a vital component in modern biotechnology.

## 1. Introduction

Plant Molecular Farming (PMF) harnesses the power of plants as bioreactors, presenting a promising avenue for the efficient production of valuable proteins, pharmaceuticals, and industrial enzymes [1,2,3,4]. Over 170 recombinant protein drugs have reached the market, targeting conditions like arthritis and cancer, with many more in development [5,6,7,8,9]. This innovative approach enables the generation of recombinant proteins through transient expression systems, plant cell-suspension culture, and stable expression. Compared to stable transformation, transient protein expression in plant cells offers a quicker and more resource-efficient alternative for developing fully transgenic plants. Utilizing various techniques such as viral vectors, agroinfiltration, hydroponic systems, and downstream processing, this method streamlines the process of gene expression in plants [3,10,11,12].

*N. benthamiana*, a plant species indigenous to the arid regions of Australia, has emerged as a central model organism in plant biotechnology and molecular farming [11,13]. Its prominence in research and production settings is largely attributed to its exceptional susceptibility to a wide array of plant pathogens, including viruses, bacteria, and fungi. This heightened vulnerability is due in part to a natural mutation in its RNA-dependent RNA polymerase gene, which impairs its antiviral defense mechanisms [14]. As a result, *N. benthamiana* serves as an ideal host for transient gene expression, a technique that allows for the rapid, temporary introduction and expression of foreign genes [15]. This makes it particularly useful for applications such as vaccine development, therapeutic protein production, and functional genomics studies, where speed and flexibility are crucial. The approval of the *N. benthamiana* plant-based COVID-19 vaccine, Covifenz, which meets Canadian safety, efficacy, and quality standards, exemplifies the regulatory achievements and underscores the global potential of transient expression systems [16].

Transient expression systems have evolved through extensive back and forth between various platforms, each offering distinct advantages and limitations. Early systems, such as the infiltration of *Nicotiana benthamiana* using *Rhizobium radiobacter* (formerly known as *Agrobacterium tumefaciens*), quickly gained popularity due to their high protein yield and scalability [17,18]. However, challenges like gene silencing and inconsistent expression led to the integration of viral vectors, such as TMV- and PVX-based systems, which improved expression levels but raised biosafety and regulatory concerns [10,19,20,21,22]. This spurred interest in deconstructed viral vectors, like the MagnICON, pEAQ, and pHREAC systems, which balanced high expression with modular design and safety [11,23,24,25,26,27,28]. More recent efforts have focused on optimizing expression timing, subcellular targeting, and co-expression of silencing suppressors to further enhance yields and functional protein recovery [25,29,30]. These iterative refinements underscore the dynamic nature of transient expression technologies as researchers continuously adapt systems to meet growing bioproduction demands.

PMF technology has improved significantly over the past decade, with yields reaching five grams per kilogram of plant biomass [31]. However, there is still a need to improve yield and downstream processing and purification, which could constitute up to 80% of total production costs. Technical and economic issues such as lignin, fibers, swelling substances, waxes, phenolic compounds, pigments, and endogenous proteases pose challenges [32]. The sheer amount of biomass poses technical and economic challenges, such as clogging chromatography columns and difficulty in removing plant pigments and phenolics from monoclonal antibody pools [33]. Other issues include scaling up filtration processes, proteolytic destruction of target proteins during downstream processing, creating an effective viral clearance method, and dealing with changing expression levels. Proper glycosylation, or “humanized” glycans, is crucial for safety and efficacy. Challenges of protein farming include public acceptance and competition with established biomanufacturing systems.

This review evaluates the strengths and limitations of various transient expression platforms in PMF and explores their considerable potential for expansion in fields such as education, synthetic biology, functional genomics, and personalized medicine.

## 2. Advancements and Applications of Plant Transient Expression Systems: A Historical Overview

**Figure 1:**
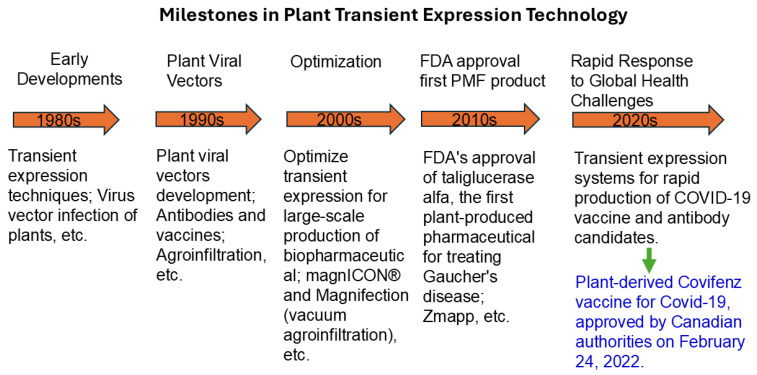
Milestones in plant transient expression technology.

The historical progression of plant transient expression technology has been defined by pivotal developments (Figure 1). The intersection of plant biotechnology and viral vectors has notably propelled advancements in this field. A significant milestone was the cloning and characterization of Cauliflower Mosaic Virus (CaMV) DNA in *Escherichia coli*, which revealed that the unencapsulated viral DNA was non-infectious in plants [34]. This finding, alongside the limitations of existing methods, helped drive the development of key DNA delivery techniques in the 1980s, such as electroporation and particle bombardment, paving the way for novel approaches to transient gene expression in plants. In a significant 1987 study, Klein et al. [35] used microprojectiles to introduce Tobacco Mosaic Virus (TMV) RNA into plant cells, successfully achieving transient expression in onion epidermal tissue. In the same decade, Marcotte et al. [36] demonstrated for the first time that a plasmid DNA containing a chimeric β-glucuronidase enzyme (GUS) reporter gene could be regulated within 60 min in rice protoplasts transformed via polyethylene glycol (PEG), enhancing transient expression technology for functional gene analysis. Concurrently, plant viruses were recognized as vectors for genetic modification, as demonstrated by the infectivity of Brome mosaic virus (BMV) cDNA in barley through in vitro assembly [37]. Additionally, Takamatsu et al. [38] documented that a TMV-based vector could produce biologically active bacterial chloramphenicol acetyltransferase in treated plant leaves ten days after inoculation.

The 1990s marked a significant era in the evolution of plant viral vectors, tailored for diverse protein productions in plant biotechnology. This decade saw the refinement of viral vector systems for specialized applications in plant science. The team led by Ow investigated the use of the Barley stripe mosaic virus (BSMV) genome for expressing foreign genes in both dicot and monocot cells, enhancing the scope of viral vectors in plant genetic engineering [39]. In a significant study by Donson et al. [40], a TMV-based vector was shown to systemically express the bacterial protein neomycin phosphotransferase across various plant systems. In 1996, Tang et al. [41] leveraged the agroinfiltration technique to transiently express the avrPto transgene in *Nicotiana sylvestris* cells harboring the *Pto* gene, inducing a defense response. This period also witnessed the application of plant virus vectors for producing various proteins, exemplified by the creation of vaccines using a Cowpea Mosaic Virus (CPMV) vector derived from *Vigna unguiculata*, and the expression of a monoclonal antibody (mAb) CO17-1A targeting a colon cancer antigen in *N. benthamiana*. Furthermore, Schob et al. [42] demonstrated the targeted delivery and expression of the P35S-CHN48 gene construct via *R. radiobacter* in *N. sylvestris*, using syringe infiltration. Notably, the CHN48 transgene was expressed exclusively in leaves transformed with the vector alone, not in those previously transformed with CHN48, which remained silent.

The 2000s marked a significant advancement in PMF with the development of various virus-based deconstructed vectors for transient expression. This innovation facilitated large-scale production of biopharmaceuticals, vaccines, and other valuable proteins, serving as an efficient alternative to traditional expression systems. It has since been widely adopted in both research and industrial applications. Key viral backbones include RNA viruses like TMV [43,44,45], Potato virus X (PVX) [46,47], Tobacco etch virus (TEV) [47], and CPMV [48], along with the DNA geminivirus Bean yellow dwarf virus (BeYDV-based vectors) [49,50,51]. Among these, the tobamovirus-based system, “magnICON^®^”, introduced by Dr. Yuri Gleba [23] and his team at Icon Genetics, achieved notable success. In 2005, Magnifection, a vacuum agroinfiltration technique, led to yields of up to 4 g of recombinant protein per kilogram of fresh leaf biomass in *N. benthamiana* and 2.5 g per kilogram in *N. tabacum* within just four days post-infiltration [52].

The FDA’s approval in May 2012 of the first plant-derived enzyme for clinical use, ELELYSO™ (taliglucerase alfa) by Protalix BioTherapeutics, underscored the potential of PMF. Utilizing carrot cells, ELELYSO™ treats adult patients with Gaucher disease, representing a pioneering achievement in plant-based pharmaceuticals [53]. Further advancements included the use of a transgenic *N. benthamiana* line lacking specific *N*-glycan residues to produce monoclonal antibodies (mAbs) with human-like glycoforms in 2011, enhancing the efficacy of an Ebola virus immunoprotective agent [54]. Additionally, a BeYDV-based vector produced an effective non-replicating subunit vaccine, Ebola immune complexes (EIC), which demonstrated protective efficacy in mice [55]. These efforts culminated in the protection of nonhuman primates using a cocktail of three tobacco-derived anti-Ebola mAbs, leading to the development of ZMapp for combating the Ebola outbreak in Africa [56].

During the 2020s, the plant transient expression platform demonstrated its potential in rapid vaccine development. Companies such as Medicago Inc. in Québec, Canada; iBio in Bryan, TX, USA; and Kentucky BioProcessing Inc. in Owensboro, KY, USA, rapidly developed VLP-or subunit-based COVID-19 vaccines within just a month [57]. Medicago’s plant-derived vaccine, CoVLP + AS03, underwent a comprehensive phase 3 trial across 85 centers worldwide, demonstrating its efficacy against various COVID-19 strains. The vaccine significantly reduced symptomatic cases by 69.5% and moderate-to-severe cases by 78.8% [58]. These results led to the approval of the vaccine by Health Canada on 24 February 2022 under the brand name “Covifenz”. Although Covifenz was not ultimately deployed in public vaccination campaigns, its regulatory approval marked a major milestone in plant-based pharmaceutical technology and significantly heightened both public and industry awareness of the potential of plants in modern vaccine development [59,60].

## 3. Plant Virus-Based Vectors in Transient Protein Expression

**Figure 2:**
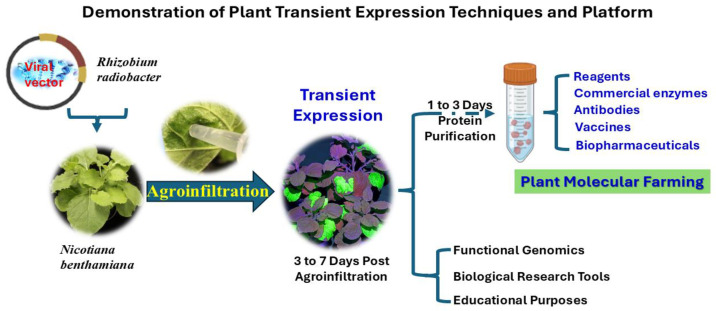
Diagrammatic illustration of the process of plant transient expression in biopharmaceuticals (PMF) and expansion.

Transient protein expression in PMF provides a rapid and efficient alternative to stable transformation, significantly shortening production timelines—from several years to just a few weeks. It also avoids complications associated with random transgene integration and the lengthy screening processes required for transgenic lines [61]. While widely used in pharmaceutical production, its utility extends into educational environments, functional genomics, and diverse biological research, enabling accelerated experimentation and data generation across these fields. In educational settings, transient expression systems offer a hands-on, accessible platform for teaching molecular biology and biotechnology, allowing students to observe gene expression, test genetic constructs, and engage with synthetic biology in real time. In parallel, their use in functional genomics supports fast and flexible analysis of gene function, protein–protein interactions, subcellular localization, and regulatory networks—all without the need for stable integration. This versatility across both learning and research contexts not only enhances education but also drives scientific discovery, positioning transient expression platforms as essential tools for both developing scientific talent and advancing genomic understanding (Figure 2).

Advanced technologies such as deconstructed viral vectors, including the pEAQ vectors from Lomonossoff’s team [62], Lindbo’s TRBO [63], the geminiviral (BeYDV) vectors developed by Mason’s group [29,64], and the magnICON^®^ systems devised by Gleba and colleagues at Icon Genetics, have been integrated with humanized glycosylation techniques in *N. benthamiana* [65,66]. These integrations facilitate rapid protein accumulation and sophisticated glycoengineering, crucial for producing high-quality therapeutics.

**Figure 3:**
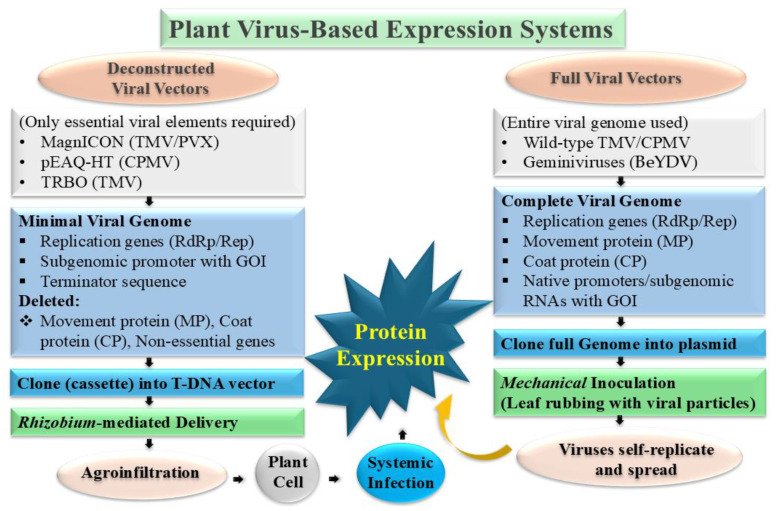
Schematic diagram of plant virus-based expression systems.

Various methods of transient gene expression are employed in PMF, using full viruses, separated viral genomes, or deconstructed viral genomes to introduce viral vector gene cassettes into plants (Figure 3). Achieving high yields is vital for the scalability and economic feasibility of PMF. Continual efforts aim to enhance production within transient expression systems. For instance, Lomonossoff’s team developed the pHREAC vector, which combines a synthetic 5′ UTR (5S0) with the 3′ UTR of CPMV, achieving a maximum recombinant protein yield of approximately 3 g/kg of GFP in fresh weight (FW) tissue, significantly higher than the 1.5 g/kg FW of GFP yields achieved with the pEAQ vector, also invented by his team [26].

Additionally, the TRBO vector, an efficient system for producing recombinant proteins, modifies the TMV genome to enable bacterial expansion. By replacing non-essential TMV genes with the target gene while preserving essential viral replication elements allows for viral replication within plant cells without the TMV coat protein gene. This adjustment boosts agroinfection efficiency and protein expression rates, with yields reaching 3 to 5 g/kg of FW, substantially increasing protein yield [63]. Essential DNA elements such as a promoter, two terminators, 5′ and 3′ untranslated regions (UTRs), and a matrix attachment region play a key role in transient gene expression. In a recent study by Coates et al. (2024) [67], the addition of extra DNA elements, specifically a 5′ UTR, to the pJL-TRBO vector enhanced GFP gene expression both in planta and at the protein level, yielding a 14-fold increase compared to the pJL-TRBO-eGFP vector [63], which did not include the UTR.

The magnICON^®^ system is one of the first transient expression platforms to successfully enter the commercial market, representing a significant advancement in PMF. This system employs *Rhizobium* to deliver viral replicons, serving as an infectious agent to facilitate the transient amplification of deconstructed viral vectors across the plant’s cellular structure [23,68,69]. The process begins when *Rhizobium* carrying T-DNA that encodes RNA replicons infiltrates the plant, setting off a systemic infection. Viral vectors then enhance cell-to-cell transmission and amplify the gene expression, achieving high-level production of recombinant proteins usually within 4 to 7 days, yielding up to 3–5 g/kg of FW in *N. benthamiana* [52,70].

The efficacy of the magnICON^®^ system in producing a range of proteins has been well documented, notably its role in synthesizing a prominent Ebola virus antibody [54,71]. Recent studies have further demonstrated its capability to produce high yields of functional foreign proteins. For example, the transient expression of recombinant human interferon α1b, α2b, and Gamma demonstrates significant biological activity against pathogens and cancer cells [72]. Variants of aflibercept equipped with specifically designed carbohydrates *N*-glycans with terminal GlcNAc (AFLIGnGn) and sialic acid residues (AFLISia) have yielded up to 2 g/kg of FW. These variants are properly assembled into dimers in glycoengineered *N. benthamiana* plants and exhibit binding potencies comparable to VEGF165 [73]. Modarresi et al. [74] successfully expressed a recombinant anti-VEGFR2 nanobody in *N. benthamiana* and *N. tabacum* cv. Xanthi, with the nanobody accumulating to 0.45% of total soluble protein, representing the first successful expression of a camelid anti-VEGFR2 nanobody in tobacco plants. Additionally, the system has been effective in producing immunoglobulin M (IgM), the largest antibody isotype known for its extensive glycosylation and oligomerization. Specific variants, H4 and P5C3 IgMs, of SARS-CoV-2 IgMs were generated in *N. benthamiana* (ΔXTFT line), yielding purified IgM at approximately 10 mg/kg and 15 mg/kg of FW for H4- and P5C3-IgM, respectively, with high glycan homogeneity. Comparative analyses revealed that, when assessing neutralizing activities, P5C3-IgM-P exhibited a roughly 12-fold increase (IC50 3 pM), and H4-IgM-P showed about a 390-fold increase (IC50 13 pM) compared to their corresponding IgG1s [75]. MagnICON^®^-based therapies have emerged as a promising platform in the realm of PMF, demonstrating both safety and efficacy in clinical and preclinical studies [76,77]. For instance, a bivalent norovirus-like particle vaccine produced in *N. benthamiana* exhibited excellent safety and robust immunogenic responses in animal models, paving the way for human trials [77].

Following the magnICON^®^ system, other viral expression vectors like the BeYDV-based vectors have further advanced the field. The BeYDV system uses a *Rhizobium*-mediated approach similar to magnICON^®^ but incorporates different viral elements (Table 1). In 2011, the Mason group engineered a high-level expression system that leverages elements from the replication machinery of a single-stranded DNA virus. This system involves the replication initiator protein (Rep), which facilitates the release and replication of a replicon from the “LSL vector”. This vector includes an expression cassette for a gene of interest, which is flanked by the virus’s cis-acting elements and was tested in tobacco NT1 cells in which the GUS expression yield that co-expressed Rep/RepA was enhanced by up to 40-fold [50,78]. A BeYDV-based single DNA replicon vector containing a built-in Rep/RepA cassette without p19 was further developed for recombinant protein expression at 0.8 g/kg FW with transient expression in *N. benthamiana* [49], demonstrating its potential for efficient and scalable protein production in plant-based systems.

The magnICON^®^ system stands as a prominent example of efficient transient expression, consistently yielding 3–5 mg of foreign proteins per gram of FW within just a week [52,70]. On the other hand, vectors based on the BeYDV have traditionally produced lower levels of recombinant proteins. However, innovative modifications to enhance these yields have shown promising results. For instance, Yamamoto et al. [79] improved the BeYDV replication mechanism by implementing a double terminator system, which integrates both a heat shock protein terminator and an extensin terminator. This modification notably increased the GFP yield to approximately 3.7 mg/g of FW within three days. Further refining BeYDV’s efficiency, Diamos and Mason [29] explored genetic modifications at the molecular level. They made a single nucleotide change in the 5′ untranslated region (UTR) of BeYDV, which successfully decreased the expression of the replication initiator proteins Rep/RepA. This alteration not only reduced cell death but also enhanced the yield of the Norwalk virus capsid protein (NVCP) to 2 mg/g of FW. These advancements indicate a significant potential for improving the productivity and application scope of BeYDV-based vectors in PMF.

The BeYDV system has undergone sophisticated optimizations to enhance its ability to efficiently co-express multiple proteins, thereby significantly improving its flexibility and scalability. These strategic advancements in the vector’s design have facilitated substantial increases in expression levels. BeYDV vectors can accommodate large genetic inserts, making them highly effective for producing high yields of monoclonal antibodies and other therapeutic proteins, with productivity increases of 4–5 times the initial yields. This system has successfully co-expressed two to four fluorescent proteins within plants, achieving notable yields of 3–5 g/kg of FW, which constitutes about 50% of the total soluble protein (TSP) [64].

Moreover, the antibodies produced using the BeYDV system, yielding 1.2–1.4 g/kg of FW, have shown strong neutralizing effects against Zika virus and other flaviviruses [64]. This demonstrates the utility of the BeYDV system in rapidly and cost-effectively producing a diverse array of pharmaceutical multi-protein complexes, highlighting its potential in advancing therapeutic solutions.

Like the magnICON^®^ system, the BeYDV system is widely used in PMF for the production of vaccines, antibodies, and various proteins in both academic and industrial settings. Rattanapisit et al. [30] demonstrated the potential of BeYDV-based vectors in *N. benthamiana* for producing the SARS-CoV-2 receptor binding domain (RBD) [80] and monoclonal antibody CR3022 using a transient expression system, with peak yields of 8 μg/g and 130 μg/g leaf fresh weight, respectively, at 3 days post-infiltration. The plant-produced RBD effectively bound to ACE2, while CR3022, though binding to SARS-CoV-2, lacked in vitro neutralization. This is the first demonstration of the *N. benthamiana*-based production of these SARS-CoV-2 proteins. In 2023, the Phoolcharoen group exhibited the efficacy of BeYDV-based vectors in *N. benthamiana* for the production of the anti-CTLA-4 monoclonal antibody 2C8. This antibody effectively binds both human and murine CTLA-4 and has been shown to inhibit tumor growth in vivo as effectively as Yervoy^®^ [81]. In the same year, their work with a purified anti-PD-L1 antibody yielded 1.8 mg/g of FW within five days post-infiltration, further demonstrating the capability to inhibit tumor growth in mice [82]. Additionally, this group reported that the BeYDV-based system in *N. benthamiana* produced a plant-purified anti-dengue virus D54 neutralizing therapeutic antibody, achieving a yield of 0.34 mg/g of FW [83]. A comparative overview of the magnICON^®^ and BeYDV systems, within the context of PMF, is detailed in Table 1, illustrating their pivotal roles in advancing biopharmaceutical manufacturing and research.

**Table 1:** A comparative overview of the magnICON^®^ and BeYDV systems in PMF.

No.	MagnICON^®^ System	BeYDV System
**1.**	The magnICON^®^ agnICON system relies on “deconstructed” viral vectors, employing *Rhizobium*-mediated systemic delivery for recombinant protein production [84].	BeYDV, a geminivirus, employs *Rhizobium*-mediated delivery to plant cells utilizing expression vectors facilitated by the viral replication initiator protein (Rep) to generate high levels of recombinant protein [78].
**2.**	Primarily functioning in tobacco, the magnICON^®^ system faces limitations in lettuce and tomatoes due to its reliance on a TMV-based viral vector [85,86].	BeYDV’s extensive host compatibility enables the efficient production of proteins at elevated levels in numerous dicotyledonous plants [87].
**3.**	Transient expression of model proteins like GFP in the magnICON^®^ system yielded 3–5 mg/g FM within one week in *N. benthamiana* [52].	Achieved exceptional yields of 3–5 mg/g leaf FW in just 4–5 days, equivalent to roughly 50% of TSP [64,87].
**4.**	The transient nature of the magnICON^®^ system, free from stable genetic plant modifications, enables faster and more adaptable production [79].	The BeYDV system offers flexibility in expressing large gene fragments and has the potential for high-volume production of recombinant proteins [88].
**5.**	Supports systemic movement and requires low *Rhizobium* density, enabling cost-effective agroinfiltration and simpler downstream processing [17].	Lacks systemic movement, required high *Rhizobium* density (OD_600_ = 0.2), leads to higher agroinfiltration costs and more complex purification due to endotoxins [29].

In plant biotechnology, choosing the right viral vector for transient expression is essential and depends on the host plant and the targeted protein. The TMV-based and BeYDV systems are known for their rapid production of high yields of recombinant proteins, typically within a week. TMV-based systems have exceptional efficacy in *N. benthamiana*, but their efficiency diminishes in other plants like lettuce and tomatoes due to species-specific interactions with TMV elements [85,86]. This limitation, which allows for the expression of only one or two genes simultaneously, restricts their application in synthetic biology, where the concurrent expression of multiple genes is often required for the synthesis of complex products.

Conversely, the BeYDV system is capable of expressing multiple genes simultaneously and managing large gene fragments across a variety of dicot plants [29,86,87]. This feature makes BeYDV highly valuable for research, gene editing, and synthetic biology. Unlike the magnICON^®^ or TRBO systems, BeYDV does not facilitate cell-to-cell movement, necessitating higher concentrations of *Rhizobium* for effective delivery (OD_600_ = 0.2) as noted by Diamos and Mason [29], compared to OD_600_ = 0.015 for TRBO as per Lindbo [63]. This leads to increased costs for agroinfiltration and subsequent endotoxin removal and purification processes, challenges that are less significant in industrial-scale settings with the TRBO system.

## 4. Comparison of Plant-Based Transient Expression Systems with Other Expression Systems

Compared to other expression platforms, plant-based transient expression systems offer a unique balance of scalability, cost-effectiveness, and the ability to produce complex eukaryotic proteins with proper folding. While they require labor-intensive infiltration processes and controlled growth conditions, they do not need sterile environments or expensive infrastructure. Plant systems typically produce plant-specific glycosylation patterns, which differ from mammalian glycosylation but are sufficient for many applications. They are particularly well-suited for medium- to large-scale production with relatively low upstream costs [61,89,90] (Table 2).

In contrast, yeast-based expression systems (e.g., *Pichia pastoris*) are easier to culture and offer faster protein production. They can perform some eukaryotic post-translational modifications (PTMs) and can be engineered to mimic human-like glycosylation, although natural patterns differ from mammalian systems. Yeast systems are robust, scalable for industrial use, and less labor-intensive but may not be ideal for proteins requiring highly specific PTMs or precise folding, where plant systems may be more favorable [91,92,93,94].

Bacterial systems, especially *E. coli*, are fast, inexpensive, and highly scalable, making them ideal for simple proteins and research reagents. However, they lack the machinery for complex PTMs and often yield misfolded proteins or inclusion bodies, limiting their use in therapeutic applications where correct folding and modifications are critical [95].

Mammalian expression systems provide the most accurate human-like PTMs and are preferred for high-end therapeutic protein production. However, they come with high costs and require sterile conditions, specialized media, and CO_2_ incubators, making them the most resource-intensive option among all systems discussed [96,97,98,99].

Insect cell systems, such as those utilizing Sf9 or Sf21 with baculovirus vectors, strike a balance between mammalian and plant systems. They support proper protein folding and more mammalian-like PTMs than plants but still require sterile conditions, bioreactors, and expensive media. While they offer higher and more consistent yields, plant-based systems remain more economical and operationally simpler for large-scale applications when exact human PTMs are not essential [100,101].

Algal systems, like *Chlamydomonas reinhardtii*, are emerging platforms known for rapid growth, low-cost cultivation, and sustainability due to photosynthetic production. They can perform some eukaryotic PTMs, but their glycosylation pathways are less defined and often incompatible with therapeutic use. Additionally, algal expression tends to produce lower and more variable yields than plant systems, which are currently more mature and reliable for producing complex proteins at scale [102].

**Table 2:** Quantitative comparison of different expression systems with key insights.

Metric	Plant-Based	Mammalian Cells	Bacterial	Yeast/Fungi	Insect Cells	Algal Systems	References
**Expression Yield (mg/L)**	1.6 g/Kg FW	3.2 g/L	1.2 g/L	1.9 g/L	0.7 g/L	1.14 μg/g	[21,103]
**Yield Notes**	High yields via agroinfiltration	HEK293/CHO provide human-like PTMs but lower yields	High yield but may lack proper PTMs	Good yields with some glycosylation	Baculovirus system allows for complex folding	Geminiviral vector allowed for the expression of recombinant proteins in *Chlorella vulgaris*	[89,103,104,105,106,107,108]
**Time to Expression (Days)**	3–7	2–10	1–3	2–5	3–4	2	[61,79,103,109,110,111,112]
**Time Notes**	Fast with agroinfiltration/viral vectors	Quick with lipid transfection, viral vectors take longer	Very fast due to simple machinery	Moderate speed for plasmid-based systems	Requires baculovirus amplification	Time varies by strain and method	[61,79,103,109,110,111,112]
**Operating cost**	Low	Very high	Low	Medium	High	Low	[113]
**Cost Notes**	Cost-effective, good for scaling	Expensive media and slow growth	Very cheap but lacks PTMs	Moderate cost with better PTMs than bacteria	Costly but allows for complex proteins	Sustainable but optimization needed	[113]
**Scalability**	High	Medium to High	Very High	High	Medium	High	[61,105,114,115,116]
**Scalability Notes**	Easily scalable, needs greenhouse/fields	Bioreactor-based, limits scale	Extremely scalable, used industrially	Optimized bioreactors allow for large-scale use	Limited by baculovirus production	Large-scale photobioreactors possible	[61,105,114,115,116]
**PTM Capability**	Moderate	High	None	Incorrect	High	Moderate	[113,117,118]
**PTM Notes**	Glycoproteins, glycosylation differs from humans	Complex, human-like PTMs	Does not support most eukarotic PTMs	Performs glycosylation but differs from humans	Provides PTMs similar to mammalian cells	Minimal PTMs, unsuitable for complex proteins	[105,119,120,121,122,123]
**Workflow Complexity**	Moderate	High	Low	Moderate	High	Moderate	[124,125,126,127,128,129]
**Complexity Notes**	Requires cultivation and agroinfiltration expertise	Needs sterile conditions and expensive media	Simple, well-established protocols	Requires bioreactor optimization for high yield	Baculovirus production adds steps	Cultivation methods need optimization	[124,125,126,127,128,129]

## 5. The Role of *N. benthamiana* and Glycoengineering for Biopharmaceutical Production

*N. benthamiana* is widely recognized as an ideal host in PMF due to its rapid growth, significant biomass yield, and high susceptibility to a variety of viral vectors. Unlike *N. tabacum*, *N. benthamiana* exhibits a complex genome with extensive chromosomal rearrangements, making it uniquely receptive to genetic manipulation [130]. Additionally, *N. benthamiana* lacks several key antiviral silencing genes, such as RDR1, RDR6, DCL2, DCL3, and AGO2, which are present in *N. tabacum*. This genetic distinction not only enhances its vulnerability to viral infections but also amplifies its efficiency as a host for viral-based expression systems [131]. Utilizing the MagnICON system, the transient expression of ER-targeted human glycoproteins like interleukin-6 (IL6) in *N. benthamiana* leaves has achieved yields as high as 7% of TSP, significantly higher than the 1% typically observed in *N. tabacum* [132]. Similarly, this system has facilitated a higher protein accumulation for potential therapeutics such as the colorectal cancer vaccine GA733-2 and the fusion protein rGA733-Fc in *N. benthamiana* compared to *N. tabacum* [133]. 

*N. benthamiana*’s rapid lifecycle, transitioning from seed to maturity in just 6–8 weeks, makes it a valuable resource for fast-track projects requiring quick production of recombinant proteins. Its ability to generate abundant leaf material enables extensive protein extraction from minimal cultivation areas, boosting its utility for both research and commercial purposes [134]. The plant’s compatibility with various viral vectors, such as TMV, PVX, and BeYDV, is largely attributed due to its compromised innate immune response. This trait facilitates efficient viral replication and gene expression, crucial for the effective use of viral vectors in transient expression systems. In addition, *N. benthamiana*’s amenability to cutting-edge genetic engineering methods, including CRISPR/Cas9 for targeted genome editing, facilitates precise genetic alterations using recombinant negative-stranded RNA rhabdovirus vectors, resulting in plants devoid of foreign DNA [135]. These attributes have significantly contributed to the deployment of various transient expression products, such as vaccines and therapeutic proteins, in industrial settings, enhancing *N. benthamiana*’s role in addressing urgent global health needs (Table 3).

### 5.1. Enhancing the Biocompatibility of Plant-Derived Pharmaceuticals Through Glycosylation Pathway Modifications in N. benthamiana

Despite extensive clinical trials showing no adverse effects from plant-specific glycosylation [136], concerns persist in the scientific community regarding the differences between plant and mammalian glycosylation pathways. To address these concerns, researchers have developed humanized glycosylated strains of *N. benthamiana* to better align with regulatory standards and enhance the biocompatibility of plant-derived therapeutics [65,66]. Advanced molecular and genome-editing techniques have enabled the precise removal of specific glycosylation markers, such as β-1,2-xylose and core α-1,3-fucose, from plant-expressed glycoproteins by targeting the respective glycosyltransferase genes for knockout in *N. benthamiana*. A significant advancement involves using a multiplex CRISPR/Cas9 strategy to create *N. benthamiana* lines devoid of both β-1,2-xylosyltransferase and α-1,3-fucosyltransferase activities (FX-KO), highlighting a move towards fully human-compatible glycosylation profiles [65]. Similarly, Jansing et al. [137] effectively used CRISPR/Cas9 to eliminate six glycosyltransferase genes, successfully producing recombinant proteins devoid of these plant-specific glycosylation elements.

Research indicates that therapeutic proteins manufactured in these genetically edited lines maintain biological activities comparable to those produced in mammalian systems, which is crucial for ensuring their therapeutic efficacy and safety [138]. Recently, the Steinkellner group engineered a marker-free genome-edited *N. benthamiana* line (NbXF-KO) that lacks seven genes associated with β1,2-xylosyltransferase and α1,3-fucosyltransferase, producing monoclonal antibodies that exhibit human-like β1,4-galactosylated and α2,6-sialylated glycosylation patterns with enhanced biological functions [139]. Furthermore, in their study, the Steinkellner team explored the glycosylation efficiency of three popular transient expression vectors—pEAQ, magnICON^®^, and pTra—within a glycoengineered plant background to express the therapeutic monoclonal antibody Cetuximab. All the vectors achieved similar, human-compatible glycosylation profiles, although slight differences in product quality were observed. Compared to the other two, magnICON^®^-based expression yielded the highest protein levels, but the antibody showed slightly increased mannosidic and incompletely processed galactosylated/sialylated structures, likely due to stress on the secretory pathway from high expression levels, highlighting both the potential and current limitations of plant-based glycoengineering to meet stringent industrial and regulatory requirements [140]. These innovations are pivotal in refining the production processes for high-quality, safe, and effective plant-derived therapeutic proteins.

### 5.2. Overcoming Downstream Processing Challenges with N. benthamiana

Downstream processing in PMF using *N. benthamiana* has traditionally been a complex and labor-intensive task, particularly in academic settings, where the extraction and purification of proteins from intricate plant matrices present numerous obstacles [141]. Various plant-derived substances such as lignin, fibers, swelling agents (like pectins), waxes, phenolic compounds, pigments, and endogenous proteases pose significant technical and economic challenges during downstream processing [142]. These components can hinder protein extraction, increase viscosity, interfere with purification, and degrade target proteins. For example, lignin and fibers make cell disruption more difficult, while phenolics and proteases can damage or inactivate the product [142,143]. Pigments and waxes complicate purification, often requiring additional processing steps [144]. Collectively, these factors increase costs, reduce yields, and necessitate specialized equipment or additives, making downstream processing a major bottleneck in the economic viability of plant-based bioproduction. Recent advancements have significantly improved both yield and purity while addressing the reduction of undesirable alkaloid content. For instance, blanching combined with chromatography simplifies the purification of the thermostable malaria transmission-blocking vaccine candidate FQS from *N. benthamiana*, achieving ~72% purity and 60% recovery while mitigating protease degradation [145]. A two-step tandem affinity purification strategy employs distinct affinity tags for each component of a protein complex, enabling the recovery of highly purified complexes suitable for structural and biochemical analyses [146]. A combined pH and temperature treatment removes approximately 70% of host cell proteins from clarified tobacco extracts and intact leaves, improving the purification of biopharmaceutical proteins sensitive to extreme conditions while broadening the applicability of precipitation-based techniques by minimizing product denaturation and loss [147]. Stephan et al. introduced a cost-effective approach involving acidic extraction and a single chromatography step, achieving a high recovery yield of purified colicins while reducing nicotine content to levels comparable to those found in common nicotine-containing foods such as tomatoes or potatoes [148]. Using ultrafiltration/diafiltration (UF/DF) with a 100 kDa membrane, Opdensteinen et al. simplified the purification of the ~11 kDa HIV-neutralizing lectin cyanovirin-*N* from tobacco extracts, achieving ~70% recovery, a threefold purity increase, and process volume reduction before chromatography [149]. Additionally, Faye et al. described a rapid and efficient method for purifying *N. benthamiana*-produced antibodies using protein A magnetic beads. This technique reduces resource requirements, operational time, and equipment needs, while delivering high-purity antibodies in a single step [150].

In industrial applications, many of these challenges have been successfully addressed, as evidenced by the commercialization of products like ZMapp™, an antibody cocktail for Ebola, and plant-based COVID-19 vaccines utilizing virus-like particles (VLPs). These achievements highlight *N. benthamiana*’s potential for the rapid, scalable, and efficient production of complex biopharmaceuticals [151,152]. Although details of industrial advancements are often proprietary, the outcomes demonstrate substantial technological progress and process optimization, ensuring consistency, efficacy, and compliance with regulatory standards.

As regulatory approvals for plant-derived pharmaceuticals grow, the role of *N. benthamiana* is set to expand further, cementing its value in swiftly and effectively addressing global health challenges. This ongoing progress in PMF technology promises more innovations and broader access to crucial biopharmaceuticals, illustrating the plant’s essential role in modern medicine and biotechnology.

## 6. Optimizing Plant Molecular Farming Through Hydroponics and Controlled Environment Agriculture (CEA)

**Figure 4:**
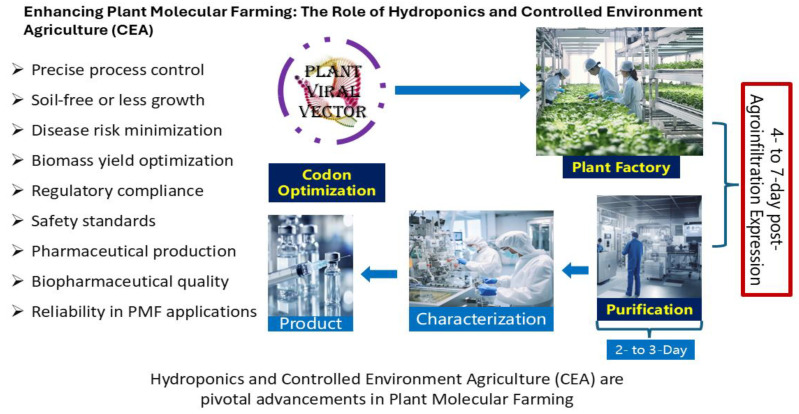
Hydroponics and Controlled Environment Agriculture (CEA) are pivotal advancements in Plant Molecular Farming (PMF).

Hydroponics and Controlled Environment Agriculture (CEA) represent significant advancements in PMF (Figure 4), providing precise control over environmental factors that critically influence plant growth and metabolic activities. Hydroponics, the cultivation of plants in a soilless medium using nutrient-rich solutions, optimizes nutrient management and water use. This method eliminates the variability associated with soil conditions and reduces the risk of soil-borne diseases, significantly enhancing the health and productivity of plants like *N. benthamiana* [1,93,152].

CEA complements hydroponics by providing meticulous control over environmental conditions such as temperature, light, humidity, and CO_2_ levels. This controlled setting allows for the fine-tuning of conditions that stimulate specific genetic pathways, enhancing the production of targeted proteins. For instance, manipulating light spectra can trigger the expression of genes essential for synthesizing specific therapeutic proteins, optimizing protein yield and quality [93,152,153,154].

These technologies not only boost plant growth and protein yield but also meet critical regulatory compliance and safety standards necessary for pharmaceutical production. The precise conditions in hydroponics and CEA ensure adherence to Good Manufacturing Practices (GMPs), pivotal for gaining regulatory approval for plant-derived pharmaceuticals. They enhance the consistency and traceability of the production process, facilitate contamination control by reducing pesticide use, and support detailed documentation of all cultivation parameters, simplifying regulatory audits.

Furthermore, these methods improve the quality control of the final biopharmaceutical product, ensuring that the proteins produced possess the necessary post-translational modifications, such as proper glycosylation patterns, which are crucial for their efficacy and safety. By streamlining these elements, hydroponics and CEA not only enhance the scalability of PMF but also reinforce its role in producing dependable and safe biopharmaceuticals, thereby supporting the expansion of plant-based drug development.

## 7. New Developments in Transient Expression Systems

Transient expression systems have undergone significant advancements in recent years, driven by the need for faster, scalable, and more cost-effective production of recombinant proteins, vaccines, and therapeutic agents. These developments encompass novel methodologies, technological tools, and enhanced host systems to improve yields, efficiency, and product quality.

### 7.1. Plant Cell Pack (PCP) System for Transient Expression: A Promising Innovation in Plant Biotechnology

Transient expression systems using *R. radiobacter* infiltration of whole *N. benthamiana* plants have been extensively explored for academic and commercial applications. However, the reliance on whole plants presents challenges, as screening multiple constructs or production conditions becomes both costly and labor-intensive. To address these limitations, the Buyel group introduced the Plant Cell Pack (PCP) system, a novel approach involving the infusion of *R. radiobacter* into three-dimensional, porous plant cell aggregates [155].

This innovative system is compatible with plant species such as *N. tabacum* BY-2, *N. benthamiana*, and Daucus carota, achieving up to a 10-times-higher efficiency than liquid culture-based transient expression methods. It supports high-throughput screening, metabolic engineering, and recombinant protein production, making it a valuable tool for process optimization. By integrating PCP preparation with automated liquid-handling systems, this method significantly reduces costs, minimizes errors, and increases throughput to over 2500 samples per day while maintaining consistent protein analysis results [156]. Notably, plant virus-derived replicating vectors have been employed in tobacco BY-2 plant cell packs, enabling scalable and efficient transient protein expression [157]. With the use of single- and multi-cassette viral vectors optimized for enhanced replication and hypertranslation, protein yields of up to ~700 ng/g fresh mass have been achieved, underscoring the potential of this system in plant biotechnology.

Despite its promise, the PCP system remains a relatively new technology. Current protein yields are still modest, and further strategies are required to improve its efficiency, yield, and scalability. Continued research and optimization efforts will be crucial to fully realize the potential of PCP-based recombinant protein production systems.

### 7.2. Transient Expression in Hairy Root Cultures

Hairy root cultures, induced by *Rhizobium rhizogenes*, provide a stable and highly efficient platform for transient expression [158]. This system combines the speed of transient methods with the robustness of root-specific expression. The advantages of this system include the reduction of costs due to continuous production in bioreactors and the enhancement of protein expression by high metabolic activity in root tissues. This system is used for secondary metabolite production and the expression of therapeutic proteins like enzymes and antibodies [158,159].

### 7.3. Bioprinted Plant Cells

The integration of 3D bioprinting with plant cells has opened new avenues for transient expression. Bioprinting creates controlled cell structures or tissues optimized for recombinant protein production [160]. The ability to produce structured tissues that enhance protein synthesis and customized scaffolds to improve cell viability and metabolic activity make this system very attractive. It finds usefulness in small-scale, on-demand protein production for diagnostics or research and prototyping novel therapeutic compounds [161].

### 7.4. Multi-Host Systems for Complex Protein Production

In some cases, transient expression systems now use co-cultures of plant cells with other organisms, like yeast or bacteria, to facilitate the production of complex proteins. This system has dual-expression systems where one host synthesizes the protein backbone and another adds post-translational modifications. Also, shared metabolic engineering optimizes yield and quality. It is used for the production of bispecific antibodies or glycoengineered therapeutic proteins [162,163].

## 8. Limitations and Future Challenges

Harnessing transient expression systems with plant viral vectors for the production of biopharmaceuticals in *N. benthamiana* has opened up new avenues for rapid and scalable manufacturing. However, despite considerable advancements, several limitations and future challenges remain.

One major limitation is the variability in expression levels among different constructs and batches. Factors such as infiltration efficiency, plant physiological state, and vector design critically influence recombinant protein yields [70,89]. This batch-to-batch variability can complicate downstream processing and regulatory approval, which demands highly consistent biomanufacturing standards.

Another significant challenge is the potential for the proteolytic degradation of recombinant proteins within the plant tissues. Host proteases may recognize and cleave foreign proteins, reducing overall yields and necessitating additional strategies such as protease inhibitor co-expression or protein engineering [164,165].

Glycosylation differences also pose a barrier for plant-produced biopharmaceuticals. *N. benthamiana* naturally adds plant-specific glycans, which differ from human glycosylation patterns and can affect therapeutic efficacy or immunogenicity [166]. Advances in glycoengineering, including the use of glycoengineered *N. benthamiana* lines, are addressing this issue, but complete human-like glycosylation is not yet fully standardized.

Scalability, while demonstrated up to several thousand liters [167], also presents technical hurdles. Large-scale infiltration requires optimized equipment and methods to maintain uniformity and avoid physical damage to plants, which could reduce yield or introduce contaminants.

Furthermore, regulatory challenges remain a critical bottleneck. Although plant-based systems offer safety advantages, such as the absence of human pathogens, regulatory frameworks are still evolving and can be complex and time-consuming to navigate [168].

Future challenges will involve improving vector design for higher and more stable expression, developing robust protease control systems, perfecting human-like glycosylation, and establishing globally harmonized regulatory pathways. Continued investment in bioprocess optimization, synthetic biology tools, and regulatory science will be crucial for the widespread commercialization of plant-made biopharmaceuticals.

## 9. Conclusions and Prospectives

Transient expression systems in plants, particularly in *N. benthamiana*, offer a rapid, versatile, and cost-effective platform for the production of biomolecules, making them essential tools in both modern medicine and scientific research. Their capacity to rapidly generate target proteins makes them especially valuable in time-sensitive scenarios such as pandemics and emerging infectious disease outbreaks. This fast turnaround has facilitated the development of modular “on-demand biofactories”, which are regional production facilities equipped with vacuum infiltration systems and controlled growth chambers, enabling agile and decentralized manufacturing of vaccines and therapeutic antibodies. These facilities provide a scalable solution to global health emergencies by ensuring localized and timely access to critical biopharmaceuticals. Moreover, transient expression systems significantly contribute to personalized medicine and synthetic biology [169,170], supporting the tailored development of treatments and enabling comprehensive genomic studies. The integration of gene editing technologies with these platforms further enhances the precision and efficiency of developing novel plant traits and therapeutic agents [171], thereby directly addressing the evolving demands of modern medicine and agriculture.

A major breakthrough enhancing the utility of this platform is glycoengineering. By knocking out plant-specific glycosylation pathways (e.g., β1,2-xylose and α1,3-fucose) and introducing components of the human glycosylation machinery, *N. benthamiana* can produce therapeutic proteins with human-compatible glycan profiles. When coupled with transient expression using plant viral vectors, this approach allows for the flexible, precise customization of monoclonal antibody glycoforms—improving pharmacokinetics and effector functions such as antibody-dependent cellular cytotoxicity (ADCC). Future advances in plant synthetic biology are expected to yield plug-and-play glycosylation modules, enabling tailored glycoprotein production for specific therapeutic targets and expanding global access to critical medications.

As transient expression systems in plants continue to mature, future innovations in downstream processing will be critical to unlocking their full commercial and therapeutic potential. The next generation of purification technologies is likely to focus on fully integrated, automated, and modular workflows that can operate seamlessly within decentralized “on-demand biofactories”. Advancements in synthetic biology may enable the design of expression constructs that incorporate smart purification tags with programmable cleavage sites, allowing for the selective release of target proteins under specific conditions. Additionally, plant lines could be engineered to express protease inhibitors or reduce host-derived contaminants, thereby improving extract purity at the source.

Beyond therapeutics, transient expression systems are influencing broader fields such as gene editing, synthetic biology, and personalized medicine. The integration of CRISPR and other gene editing technologies enhances the precision and efficiency of engineering both plant traits and therapeutic agents. These systems also support rapid prototyping and customization of treatments, aligning with the demands of personalized medicine. Educational applications are expanding as well; for instance, the EU Horizon 2020 Pharma-Factory consortium has developed a freely available Plant Molecular Farming (PMF) kit (https://www.ispmf.org/lab-protocols accessed on 18 May 2025) to facilitate hands-on learning, allowing students and researchers to quickly explore gene function and molecular interactions.

Looking forward, the integration of artificial intelligence and digital agriculture will further optimize plant-based bioproduction. Smart growth chambers equipped with AI-based monitoring and predictive analytics will enable dynamic control of environmental variables to maximize protein yield and quality. While *N. benthamiana* remains the preferred host for PMF, researchers are exploring alternatives like lettuce 60, which offers advantages such as lower levels of phenolics and alkaloids and compatibility with hydroponic cultivation. Developments in plant glycoengineering expand the range of biopharmaceuticals that can be produced, increasing global access to crucial medications. As these technologies continue to evolve, their potential to meet significant healthcare and agricultural challenges is substantial, paving the way for groundbreaking biotechnological applications.

## Figures and Tables

**Table 3:** List of companies using *N. benthamiana* for plant transient expression products.

Company Name	Country	Product	Vector System Used	Website/Reference
**Icon Genetics (Denka)**	Germany	Various biologics	magnICON^®^ system	https://www.icongenetics.com/
**Kentucky BioProcessing (KBP)**	USA	Vaccines, antibodies	TMV, other vectors, magnICON^®^ system	https://kbio.com/
**Leaf Expression Systems**	UK	Proteins, antibodies, enzymes	Hypertrans^®^ Expression System	https://www.leafexpressionsystems.com/
**PlantForm Corporation**	Canada	Antibody drugs, biosimilars	vivoXPRESS^®^	https://www.plantformcorp.com/
**Cape Biologix**	South Africa	Therapeutic proteins	PtX™ viral system	https://capebiologix.com/
**Baiya Phytopharm**	Thailand	Vaccines, antibodies, growth factors	BaiyaPharming™	https://baiyaphytopharm.com/
**Nomad BioSciences**	Germany	Antibacterial and antiviral biologics	magnICON^®^ and NOMADIC™ platforms	https://www.nomadbioscience.com/
**Bioapplications**	South Korea	Vaccines	magnICON^®^ system	https://www.bioapplications.global/ (accessed on 18 May 2025)
**Cirsium Biosciences**	USA	AAV vectors	TMV, other vectors, magnICON^®^ system	https://cirsiumbio.com/
* iBio Inc.	USA	Vaccines, therapeutic proteins	Hypertrans^®^ Expression System	https://ir.ibioinc.com/
* Medicago	Canada	Vaccines (e.g., COVID-19 vaccine)	vivoXPRESS^®^	[59]

Note: * Medicago has ceased operations. iBio is accelerating its transformation into AI-powered biotech company.
